# Partners in Care: Training Healthcare Professionals in Using Patient Feedback

**DOI:** 10.1111/tct.70130

**Published:** 2025-06-25

**Authors:** Matthijs H. Bosveld, Max Hermans, Marcel Verhoeven, Frank Smeenk, Carolin Sehlbach

**Affiliations:** ^1^ Care and Public Health Research Institute (CAPHRI), Department of Family Medicine Maastricht University Maastricht The Netherlands; ^2^ School of Health Professions Education (SHE), Faculty of Health, Medicine and Life Sciences Maastricht University Maastricht The Netherlands; ^3^ Patient as a Person Foundation Eijsden The Netherlands; ^4^ Catharina Hospital Eindhoven The Netherlands

## Abstract

**Background:**

Patients are the *raison d'être* of all those working in healthcare. Despite the recognised value of patient feedback, its integration into healthcare professionals' lifelong learning remains limited. At the same time, healthcare professionals have most of their contact with patients after completing their training, and with it, the biggest impact on patient care. We present the development and evaluation of an interprofessional, evidence‐informed patient feedback training activity for healthcare professionals.

**Approach:**

Using a design‐based research approach, we conducted interviews with 12 healthcare professionals and 10 patients to explore perspectives on patient feedback and lifelong learning. Insights from these interviews informed the design of a two‐session training program, which we piloted in an academic hospital and evaluated through observations and a survey. The first training session covers theoretical aspects of and patients' lived experiences with patient feedback. In‐between sessions, healthcare professionals are tasked with gathering patient feedback in daily practice. In the second session, participants discuss experiences and engage in peer‐to‐peer coaching facilitated by a trained patient.

**Evaluation of Innovation:**

The training fostered a safe space for open dialogue between patients and healthcare professionals. Professionals sharing personal accounts on their own lived experience with care helped participants recognise healthcare professionals' humanity and highlighted the value of patient feedback in professionals' continuing development.

**Implications:**

Engaging patients and healthcare professionals in co‐designing training programs supports healthcare professionals' use of patient feedback. While trainings can help professionals incorporate patient feedback into their learning, genuine integration requires embedding feedback‐seeking behaviours into daily practice.

## 1 Background

Patient feedback is increasingly used in undergraduate and postgraduate education of healthcare professionals, including interprofessional education [1, 2]. In many cases, the active involvement of patients^1^ in education provides multiple benefits. On the one hand, students benefit from patient and public involvement. Through patient feedback, their education becomes more transformative and engaging [3, 4]. On the other hand, patients benefit from their participation in education. Patients gain understanding of their own condition, become more assertive in contact with health professionals, and feel valued and empowered [5].

Healthcare professionals have most of their contact with patients after completing their training, and with it, the biggest impact on patient care. This creates a rationale for the integration of patient feedback in continuing professional development.

Patient feedback can help healthcare professionals (HCPs) to identify blind spots, and inclusion of patient perspectives in lifelong learning can result in a more effective learning environment [6]. Despite these known advantages and the acknowledgment of the importance and transformative character of patient feedback in workplace learning, the role of patient feedback in lifelong learning of healthcare professionals is still relatively small [7].

This is partly due to conflicting roles experienced by professionals as either expert or learner as well as a subsequent changes in the physician–patient relationship and power dynamics. Issues around power in the relationship, vulnerability and development of professional identity also hinder the use of patient feedback [8]. Lastly and most importantly, patients and professionals often lack the tools to engage in meaningful feedback conversations in practice [8]. Therefore, the goal of our innovation was to develop a training which aims to facilitate the invitation and use of patient feedback for healthcare professionals' lifelong learning.

## 2 Approach

**FIGURE 1:**
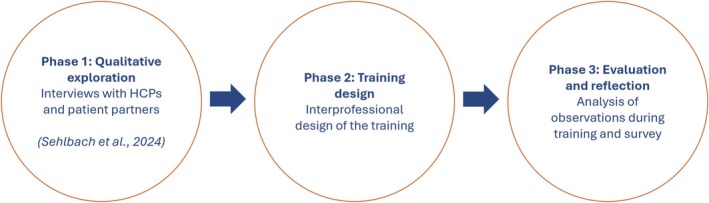
Overview of the design‐based research approach.

The authors undertook a design‐based research (DBR) approach (Figure 1). DBR studies take place in real‐life settings, use multiple methods, and are characterised by continuous cycles of design, evaluation, and redesign to advance theory [9]. In phase 1, HCPs and patients were interviewed on their perspectives on (engaging patients in) feedback conversations with their patients or provider as informal learning opportunities. To summarise the outcomes of these interviews, previously published [8], both patients and HCPs acknowledged the importance and value of patient feedback. However, participants indicated that they lack competencies and strategies to engage in patient feedback conversations in practice.

In phase 2, based on the outcomes of the interviews, the *Partners in Care* training program was designed. The target group for the training program entails a broad range of HCPs (physicians, nurses and physicians or nurses in training). A *patient partner* (MV) was part of the design team. A *patient partner* is someone with experiential expertise in the field of health care and/or social services, who can transcend personal experiences to contribute constructively to person‐centred care in the domains of health care and social service. In this instance, the patient partner completed the Patient as a Person‐academy program [10]. Additionally, the team consisted of two clinicians (FS and MB), one clinician in training (MH), two researchers (CS) and one student assistant. We organised two design sessions to discuss the structure and content of the training program. These discussions were informed by the interviews conducted as well as the combined experience and expertise of the authors and implicit assumptions based on Kolb's experiential learning theory [11, 12]. The input from the *patient partner*, researchers, and clinicians enabled a training program in which all respective actors have a complementary part to play.

**FIGURE 2:**
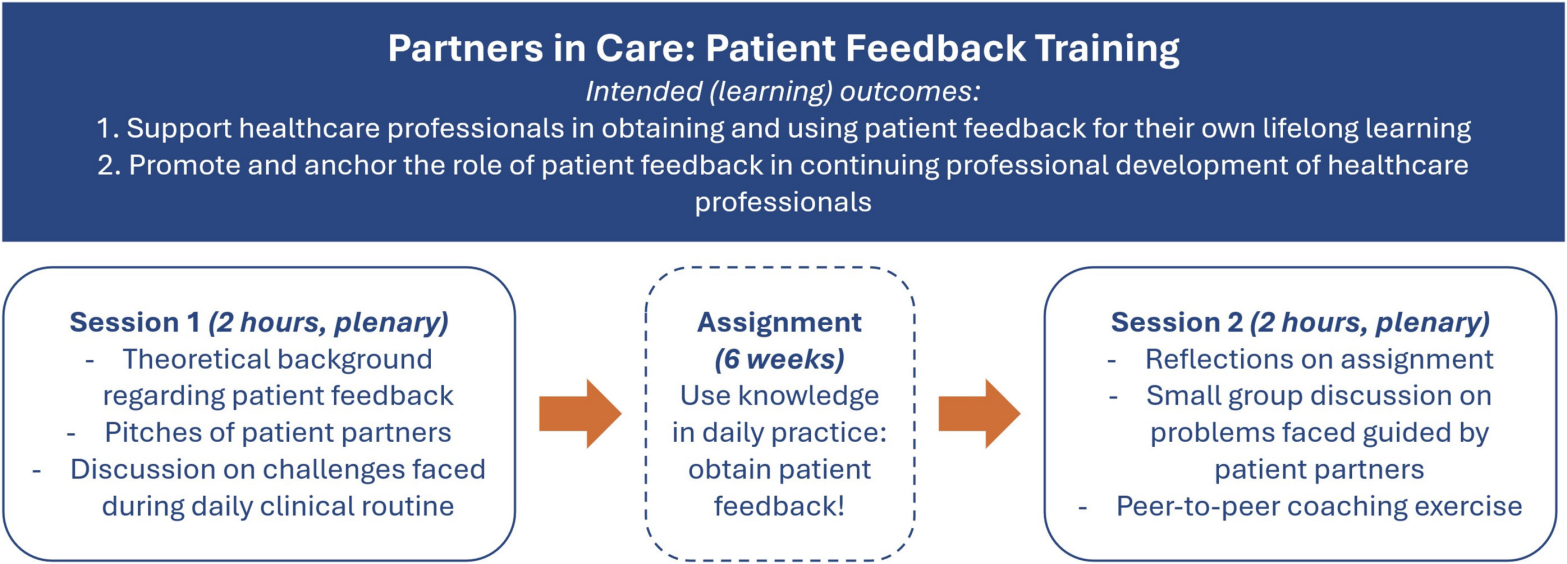
Overview of partners in care: patient feedback training.

The resulting training consists of two 2 h meetings, approximately 6 weeks apart (Figure 2)—an example in which complementary expertise came into play to navigate logistical challenges. Patients and professionals did not want a meeting longer than 2 h, patients preferred a training during the day due to energy constraints, and HPCs needed to have enough patient interactions in between the sessions. *Patient partners* are present, as well as two faculty members during both meetings, to facilitate the sessions and provide feedback on the experiences of participating HCPs.

The first session focuses on the theoretical background of patient feedback, and two *patient partners* share personal experiences with giving patient feedback or experiences in which they felt the urge to give feedback—but did not. The aim is to prepare and equip participating HCPs to elicit patient feedback in their daily practice to prepare for the next training session. To facilitate this, *patient partners* pitch their experiences *en groupe* and interactively lead smaller subgroups in which participants react to these pitches and reflect upon them. At the end of the first session, a hand‐out with useful tips (Appendix S1) for receiving feedback in daily practice was provided as hand‐on support for participants [13].

The second session aims to discuss experiences in gathering patient feedback, peer‐to‐peer coaching on these experiences in small groups, and articulation of learning goals for continuing development.

In phase 3, the training program was piloted and evaluated at Maastricht University Medical Centre. In this academic hospital, all nurses and physicians (in training) can register to a variety of training activities as part of the institutional interprofessional education, including the Partners in Care training. These training activities focus on the development of generic competencies as part of employees continuing professional development. Evaluation of this training took place by realist observation of the training sessions by a non‐participating observer using a semi‐structured observation guide (Appendix S2) and a brief survey (Appendix S3) [14]. Realist observation does not entail just ‘watching what happens’, it regards the theory‐informed observation of the context‐learning interplay. A semi‐structured observation guide aids the observer to identify relevant interactions during the pilot of the training.

## 3 Evaluation of Innovation

The observation of the sessions focused on the atmosphere and interaction between participants and *patient partners*. There were seven participants in the first session, six in the second. Participants differed in professional background and experiences, representing (specialised) nurses, residents and medical specialists. The faculty members mainly had experience in or affinity with a clinical setting, but the available interprofessional expertise was deemed complementary, well‐balanced and seen as a strength in the preparation and execution of the training.

Analysis of the observational data indicated two important aspects of the training: (1) open interaction between patients and HCPs and (2) the healthcare professional as a human. Regarding the first aspect, HCPs and patients both shared thoughts and feelings of various personal experiences during the training sessions. They discussed different viewpoints and reacted to each other's stories, becoming equal partners.

**Excerpt 1:** A *patient partner* shares a personal story on a visit to an orthopaedic surgeon for shoulder pain. They discussed the outcomes of diagnostic imagery, after which the orthopaedic surgeon concludes: ‘There is nothing wrong with your shoulder’. The patient partner expressed her devastation by this news, as the pain in her shoulder is real. The surgeon replies: ‘It was wrong of me to put it that way, I should have said: the magnetic resonance imaging does not show the cause of your shoulder pain’.
One of the participating nurses responds: ‘I think it's very brave of you to speak up to this to the doctor, to address the doctor's communication skills, walking away would have been easier’.

In this excerpt, the nurse compliments the *patient* *partner* and practices perspective taking by understanding the difficulties patients might face in giving feedback in their interactions with healthcare professionals [15].

During these observations, we furthermore noticed that almost all participants shared a personal story of being ill or a close relative being ill. By showing vulnerability as HCPs, they influence the relationship among themselves and between patients, subsequently influencing (power) dynamics.

**Excerpt 2:** A participating nurse shares a personal story about being hospitalised and not getting pain medication after pressing the alarm. After waiting for a long time, she decides to call the department through her cellphone as she had previously worked there. She asks her colleague for pain medication, receives it, and upon reflection shared: ‘What if I was a normal patient who did not know this number? Would I have to suffer for several hours until they remembered me?’

By narrating these personal stories about uncertainty and dependency, HCPs showed their human side as patients instead of professionals. Altering or switching between different roles (from patient to provider and vice versa) that happened during the training was deemed valuable by all participants. Despite its value, participants agreed that due to time limitations and daily routines these reflections rarely happen during daily practice.

In the survey, participants described the training as inspirational and helpful. Participants differed in their preferences for the depth of the theoretical background and wanting to practice eliciting and discussing patient feedback more during the sessions. The interprofessional aspect of the training was seen as a strength of the training, including HCPs of various disciplines and experience levels. Furthermore, the collaborative aspects of *patient partners* participating in the sessions were deemed a positive asset of the sessions by the participating HCPs.

This study has several limitations. The first is that the observation guide was discussed within the research team, but was not piloted. Furthermore, the evaluation was restricted to the evaluation of participants' perceptions, limiting the conclusions to be drawn regarding its impact on patient care.

## 4 Implications

Engaging patients, researchers and HCPs in co‐designing training programs supports and enables healthcare professionals' use of patient feedback. By positioning patient partners and HCPs as equal partners, power differences in the relationship seemed to fade during the training and room for vulnerability from both patient partners' and HCPs' side were observed. Future research initiatives should not only evaluate perceptions of participants but also explore the clinical impact of such training initiatives on outcomes such as behaviours or experience of care—across various contexts in the global north and south.

The *Partners in Care*‐training program marks a valuable step towards a continuum of active patient involvement throughout the education of healthcare professionals: preferably starting in undergraduate and postgraduate education and anchored in continuing professional development. This aligns with tips that Eijkelboom et al. share on how to achieve greater patient involvement in health professions education—start from day one and never stop [3]. While training can help HCPs incorporate patient feedback into their learning, genuine integration requires embedding feedback‐seeking behaviours into daily practice—by the learner and by the organisation. The latter could facilitate this integration through, for example, the provision of additional time for or training on eliciting patient feedback in the workplace or underscore its importance in reaccreditation standards for healthcare professionals.

## 5 Conclusion

The current training program is focused on healthcare professionals' competencies to elicit and use patient feedback. However, optimal use of patient feedback not only requires not only physicians, but also patients. Future initiatives should focus on raising awareness that patients may possess the power to guide healthcare through their feedback, as well as supporting patients in providing tailored feedback beyond regular questionnaires.

## Conflicts of Interest

Marcel Verhoeven is director of the Patient as a Person Foundation.

## Supporting information


**Appendix S1** Hand‐out with tips on asking patient for feedback.

**Appendix S2** Semi‐structured observation guide.

**Appendix S3** Evaluation questionnaire.

## Data Availability

The data that support the findings of this study are available from the corresponding author upon reasonable request.

## Endnotes

^1^
We use the term ‘patient’ to be inclusive of service‐users, consumers, carers and/or family members although the important distinctions between these terms is acknowledged.
