# Supplementary material for: Partners in Care: Training Healthcare Professionals in Using Patient Feedback
Source: Clin Teach. 2025 Jun 25;22(4):e70130. doi: 10.1111/tct.70130 (PMC12188500; doi:10.1111/tct.70130)
Supplement: Supplementary file 1 — Appendix S1 Hand‐out with tips on asking patient for feedback. [file TCT-22-e70130-s003.docx]

**Appendix I – Semi-structured observation guide**

**General**

- Which training is being evaluated?
- What is the set-up of the room?
  - Who (which healthcare professional) is sitting where?
- How many participants are present?
- How many research team members are present?

**Atmosphere**

- What is the atmosphere like?
  - How does the program leader create a safe environment to stimulate discussion?
- How do participants react to shared experiences?
  - Concrete examples
  - Verbal and non-verbal cues/reactions
- How does the atmosphere change during the patient pitch? In what way? How do participants react on the patient pitch?

**Interaction**

- Is there a discussion on patient feedback? Who is leading this discussion? Who participates in this discussion?
  - Patients, nurses, residents, medical specialists?
  - Who is asking what, when?
- How do participants interact among each other? Do participants share experiences/emotions?
- Are their boundaries between healthcare professional and patient partners? In what way? How is this visible?
- What is the role of the patient partners during the sessions? Do participants/healthcare professional involve them (patients)?

**Evaluation**

- What are feedback points from participants? How do other participants think about this (agree/disagree)?
- What are things to maintain?
- What are things to improve upon?
