# Supplementary material for: Partners in Care: Training Healthcare Professionals in Using Patient Feedback
Source: Clin Teach. 2025 Jun 25;22(4):e70130. doi: 10.1111/tct.70130 (PMC12188500; doi:10.1111/tct.70130)
Supplement: Supplementary file 2 — Appendix S2 Semi‐structured observation guide. [file TCT-22-e70130-s001.docx]

**Appendix II – Evaluation questionnaire**

1. Which aspects did you appreciate in this training?
2. What is the main “take-home message / eye-opener” for you? Or what is the direct applicability in your daily routine?
3. Did you miss anything during the training, for example, specific information, exercises or advise?
4. What can be changed to improve this training?
5. What did you like about the trainers, and do you have any tips for them?
