# Supplementary figures and images for: Partners in Care: Training Healthcare Professionals in Using Patient Feedback
Source: Clin Teach. 2025 Jun 25;22(4):e70130. doi: 10.1111/tct.70130 (PMC12188500; doi:10.1111/tct.70130)

**Appendix III – Hand-out with tips on asking patient for feedback
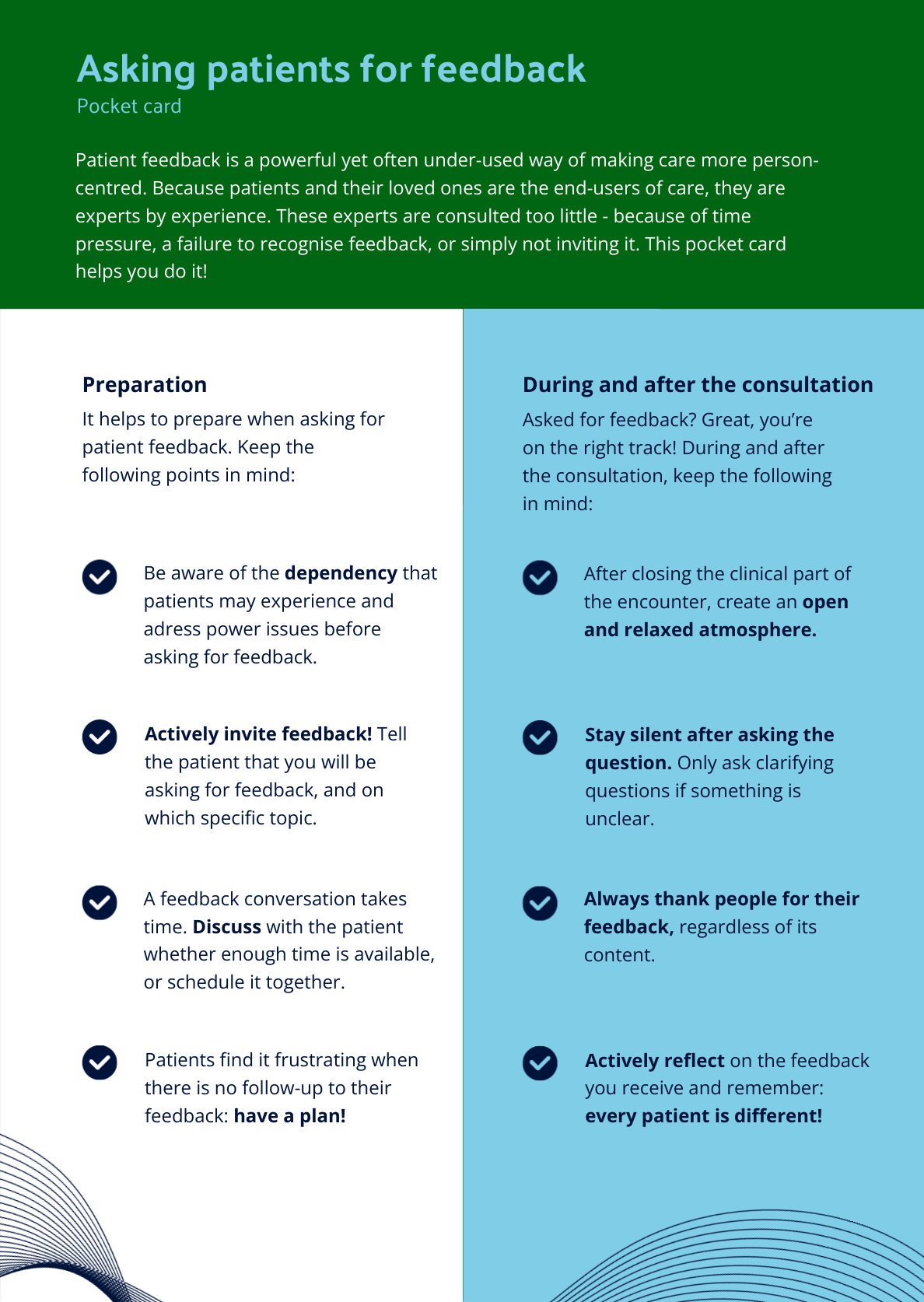

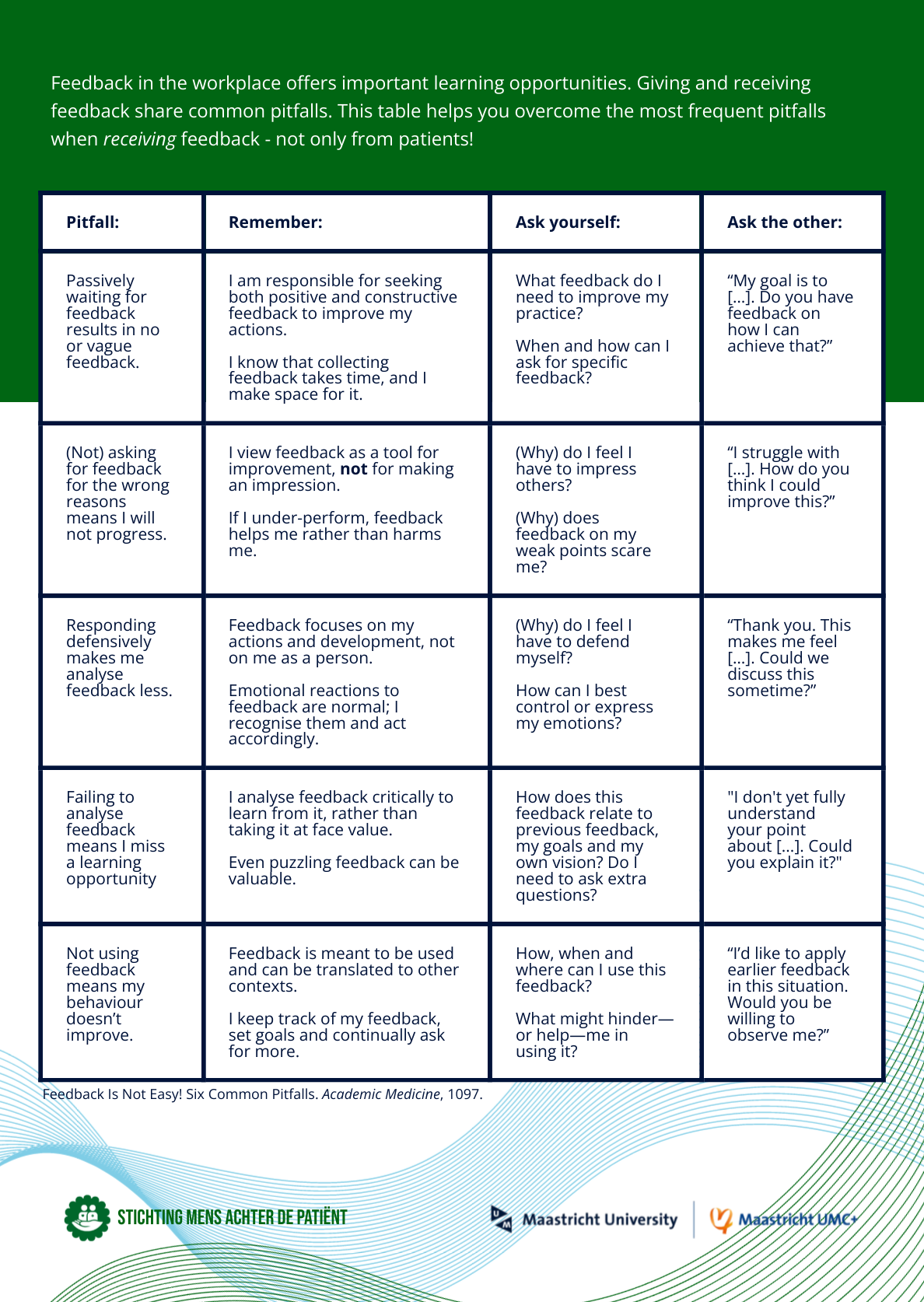
**

Supplement: Supplementary file 3 — Appendix S3 Evaluation questionnaire. [file TCT-22-e70130-s002.docx]
